# Polymorphism of exon 13 lgr8 gene as risk factors for cryptorchidism in children

**DOI:** 10.1186/1687-9856-2013-S1-O26

**Published:** 2013-10-03

**Authors:** I Wayan Bikin Suryawan, Soetjiningsih, Ketut Suastika, Joserizal Latief batubara

**Affiliations:** 1 Faculty of Medicine, Udayana University, Bali, Indonesia; 2 Faculty of Medicine, University of Indonesia, West Java, Indonesia

## 

Cryptorchidism refers to incomplete descend of the testes into the scrotum with the testes located in the normal tracts. The incident is 3-5% among infants born at term. The complications are infertility and testicular cancer. Cryptorchidism has a multifactorial aetiology and the role of exon 13 lgr8 gene polymorphism remains unclear.

This study aims to find the frequency polymorphism of exon 13 lgr8 gene in children with cryptorchidism and to prove as a risk factor for cryptorchidism.

This study is an observational case control study, conducted at the children’s in two general hospital and one maternity clinic from September 2010 until March 2011, with 31 children cryptorchidism as cases and 31 healthy children as controls who were matched by gestational age and age of the child. Polymorphism of exon 13 lgr8 gene was evaluated by sequencing of PCR results at YAYASAN GENEKA Eijkman Molecular Biology Institution Jakarta. Frequency was analyzed by percentage, polymorphism was analyzed by paired odds ratio and analyzed by computerized programmes, hypothesis with a confidence interval (a) was accepted if p<0,05.

This study found a percentage of intraabdominal cryptorchidism of 41,94%, inguinal cryptorchidism of 41,94%, and prescrotal cryptorchidism of 16,12%. This study also found a frequency polymorphismof exon13 lgr8 gene (S337A = 54,84%; P340P = 41,94%; H345P = 61,29%; K346K = 90,32%; Q354K = 16,13%; Q356P = 29,03%; S357S = 48,39%). Polymorphisms S337A (TCA→ GCA) p = <0,001; H345P (CAC → CCC) p = 0,03; Q356P (CAG → CCG) p = 0,004; and S357S (TCT → TCC) p = <0,001 of exon 13 lgr8 gene were found significantly more often in cases.

In summary polymorphism S357S is associated with a new marker; polymorphisms S337A; Q356P are associated with an increased risk; polymorphism H345P is associated with an increased risk or new marker of cryptorchidism in boys. This study is expected to be used in the determination of therapeutic decisions, as a guide for prognosis and as a foundation for further studies.

